# Force Analysis and Evaluation of a Pelvic Support Walking Robot with Joint Compliance

**DOI:** 10.1155/2018/9235023

**Published:** 2018-11-28

**Authors:** Jiancheng Ji, Shuai Guo, Fengfeng (Jeff) Xi

**Affiliations:** ^1^Department of Mechatronic Engineering and Automation, Shanghai University, Shanghai, China; ^2^Department of Aerospace Engineering, Ryerson University, Toronto, Canada

## Abstract

The force analysis of a pelvic support walking robot with joint compliance is discussed in this paper. During gait training, pelvic motions of hemiplegic patients may be excessively large or out of control; however, restriction of pelvic motions is not likely to facilitate successful rehabilitation. A robot-assisted pelvic balance trainer (RAPBT) is proposed to help patients control the range of motion via force field, and force analysis is necessary for the control of the compliant joints. Thus, kinematic model and static model are developed to derive the Jacobian and the relation between the interaction forces and the pelvic movements, respectively. Since the joint compliance is realized through a nontorsional spring, a conventional (linear) Jacobian method and a piecewise linear method are derived to relate the interaction forces with the pelvis movements. Three preliminary experiments are carried out to evaluate the effectiveness of the proposed methods and the feasibility of the RAPBT. The experiment results indicate that the piecewise linear method is effective in the calculation of the interaction forces. Gait with pelvic brace strongly resembles free overground walking and partly decreases motion range via force field. The findings of this research demonstrate that the pelvic brace with joint compliance may provide effective interventions.

## 1. Introduction

With the aging of the population, most stroke survivors are suffering considerably from a loss of physical mobility. To improve the walking function of elderly people and stroke patients, lower limbs rehabilitation robots have been developed to assist patients for gait rehabilitation [1], such as Locomat [2], LOPES [3], ALEX [4], and Lokohelp [5] are treadmill-based exoskeleton robots with body weight support (BWS) system, which provide powered assistance at the hip and knee. However, the principle of these lower limbs rehabilitation robots is to simply utilize the end-effector for the attachment of pelvis [5]. One problem associated with this design is the misalignment between the rotation center of the end-effector and the actual rotation center of the pelvis. Without joint compliance, the force generated by the pelvic motions cannot be absorbed by the robot and will react back to the patient. This interaction force could cause injury, which is often referred to as the secondary damage [6].

Furthermore, treadmill-based exoskeleton robots restrict pelvic motions that can lead to less satisfactory functional outcomes after intervention [7], and pelvic rotation, pelvic tilt, and lateral displacement of pelvis (key gait motion parameters) are related to pelvic motion, which emphasizes the importance of pelvic support mechanism [8]. Therefore, the fixation of pelvis should be avoided to obtain more realistic locomotion and natural gait.

To solve this problem, several pelvic support robots have been developed. For example, a pelvic support robot based on functional electrical stimulation (FES) was developed by Waseda University [8], but control of FES needs further studies; an omnidirectional robotic system with passive body weight support was developed by Carleton University [9], and it can reduce pelvis and torso motion constraints, but the effect on balance training for patients needs investigation; pelvic support mechanism for dynamic balance training was studied by Matjaž et al. [10], and they replaced the passive pelvic structure with linear actuators, so that all pelvic motions can be satisfied. Furthermore, overground walking platform with pelvic support mechanism, such as KineAssist [11], RPB [12], BAR [13], and NaTUre-gait [14] are mobile robotic systems for gait training, which enable natural gait with proper sensory input.

**Figure 1:**
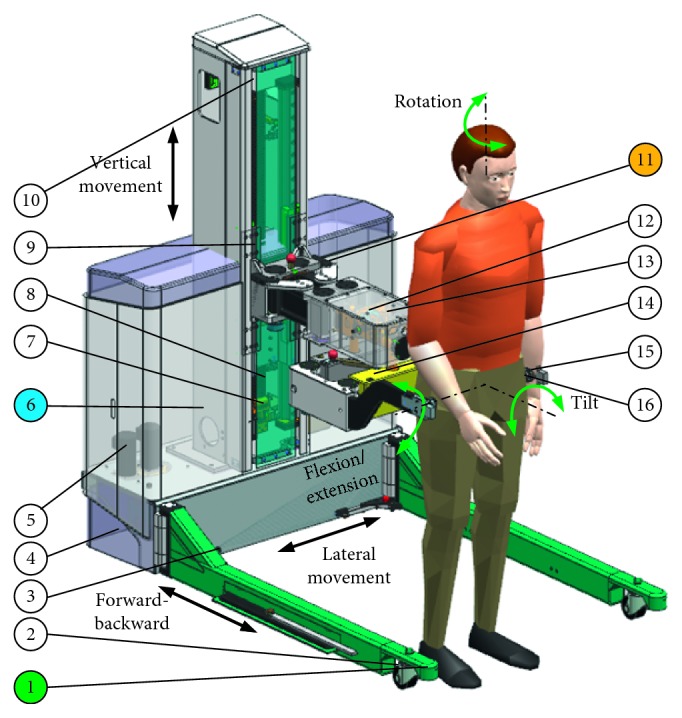
RAPBT-conceptualized design and the prototype of the trainer. Detailed mechanical design of robot-assisted pelvic balance trainer is given. Omnidirectional mobile platform (OMP): 1, mobile platform frame; 2, castor wheels; 3, deployable mechanism; 4, drive motors; 5, control unit (Beckhoff PLC CX5130). Partial body weight support system (PBWS): 6, PBWS frame; 7, tensioning wheel; 8, synchronous belt; 9, linear guides, 10, servo motor (BMH1401P11A1A). Pelvic brace (PB): 11, pelvis element; 12, encoders; 13, springs; 14, adjusting mechanism; 15, pair of ATI F/T sensors (Mini 45); 16, spherical joint.

In this paper, a novel pelvic support robot (shown in Figure 1) with BWS is presented, and this robot consists of an omnidirectional mobile platform, a pelvic support mechanism containing a body weight support system, and a controller. The main principle of this robot is based on the passive compliance embedded in the robot joints. Discussed in this paper is the force analysis required for the development of this robot. In what follows, details are provided.

## 2. System Description

**Figure 2:**
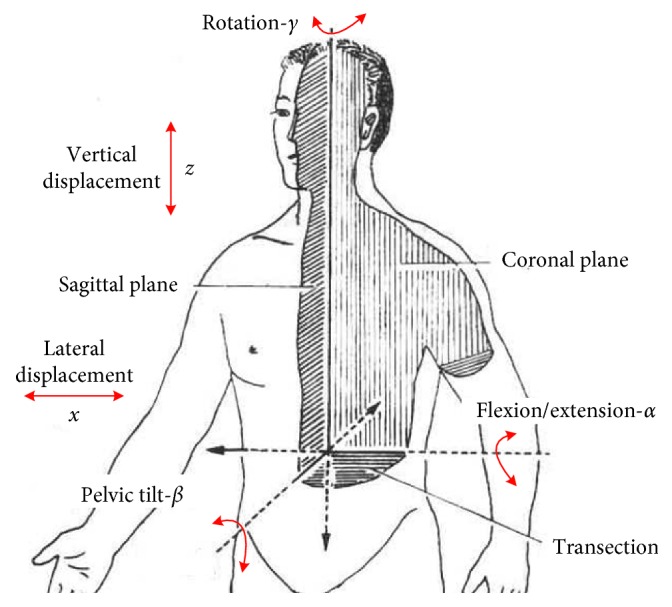
Pelvic DoFs. Available DoFs in pelvis when walking within RAPBT. Actuated DoFs: actuated forward/backward movement, actuated lateral movement, and actuated vertical movement. Passive DoFs: passive left/right displacement, passive tilt, passive rotation, and passive flexion/extension.

As shown in Figure 2, six degrees of freedom (DoFs) of pelvis can be satisfied when walking with RAPBT: actuated forward/backward movement, lateral movement, vertical movement and passive mediolateral displacement, pelvic tilt, pelvic rotation, and flexion/extension. The movements of the patient have the characteristics of high amplitude, asymmetry, and aperiodicity. Therefore, a pelvic support robot should meet all motion requirements with joint compliance to avoid rigid impact and secondary damage.

The overall conceptualized design of the trainer is shown in Figure 1, the RAPBT system consists of (i) an omnidirectional mobile platform (OMP); (ii) a partial body weight support system (PBWS); and (iii) a pelvic brace (PB) [15]. The primary aim of OMP and PBWS is to provide overground mobility and body weight support, respectively, and to ensure rigid support basis and appropriate attachment locations for the PB. With the RAPBT, subjects can move in any direction without being constrained over ground and no limitation for pelvis so that the therapist can facilitate the gait rehabilitation and balance training more effectively.

OMP is designed as a U-shaped rigid steel frame with deployable mechanism, to provide patients approximately 0.9 m of free space in medio/lateral direction (*X*-axis) and approximately 1.2 m of free space in anterior/posterior direction for unrestricted foot placement and 360° rotational motion during gait. OMP is supported with two castor wheels that enable angular motion of the OMP and two drive omnidirectional wheels that are installed at the back so that center of mass (CoM) of the body is simultaneously shifted on the ground. The PBWS connected with the OMP is designed to realize the approximately 0.6 m vertical displacement (*Z*-axis) of pelvis and provide subjects appropriate body weight support via a synchronous belt and a set of linear guideways, labeled by *R*_0_.

**Figure 3:**
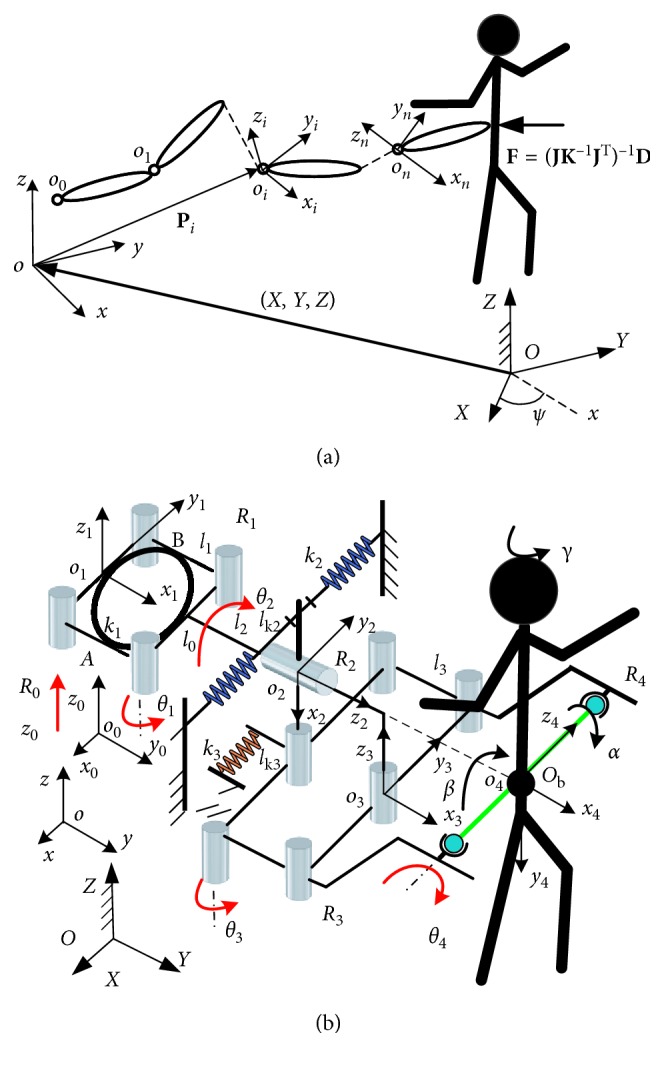
Kinematic scheme of RAPBT and reference frames of PB.

As shown in Figure 3, pelvic brace (PB) consists of a parallelogram four-bar mechanism labeled by *R*_1_, a pendulum mechanism labeled by *R*_2_, a parallelogram six-bar mechanism labeled by *R*_3_, and pair of spherical ball joints labeled by *R*_4_. *R*_1_ is used to realize small displacement along the *X*-axis or *Y*-axis during gait. As shown later in the paper, this joint can be simplified as a revolute joint. It should be noted that the large movement in the *X*-axis or *Y*-axis will have to be realized by OMP. *R*_2_ is used to realize the pelvic tilt about the *Y*-axis. This is a long revolute joint with its axis in parallel to the *Y*-axis, with a rotational range of ±20°. *R*_3_ is used to provide a rotation around the Z-axis with an angular range of ±25°. *R*_4_ consists of two spherical joints that are used to connect the pelvis and realize the rotation around the *X*-axis, i.e., bending. Furthermore, a set of adjusting mechanism is designed to accommodate for different sizes of patients. In our design, OMP and PBWS are motorized using five motors, whereas *R*_1_, *R*_2_, and *R*_3_ are passive and embedded with compliance.

Figure 3 shows the kinematic scheme of RAPBT and reference frames of PB, and the compliance of *R*_1_ is realized by a circular leaf spring that is installed inside of the parallelogram mechanism. When the parallelogram rotates, it deforms the leaf spring to generate a reset force against the rotation of *R*_1_, thereby providing a joint compliance. The compliance of *R*_2_ is accomplished by two linear springs that are installed in the lateral direction on both sides of the pendulum mechanism. When *R*_2_ rotates, the two springs deform to generate spring forces against the rotation of *R*_2_, thereby providing a joint compliance. The compliance of *R*_3_ is created in the same way as that of *R*_2_. In addition, encoders are installed in the three joints to measure the joint angles that can be used to determine the joint forces if joint stiffness is known.

Kinematically, PB can be viewed as a serial-parallel hybrid mechanism with four joints. As explained before, *R*_1_ and *R*_3_ are two revolute joints realized by respective parallelogram mechanisms, and their movements are denoted by *θ*_1_ and *θ*_3_. *R*_2_ is a conventional revolute joint and its movement is denoted by *θ*_2_. *R*_4_ is only used to connect with the patient pelvis which is represented by a link between the two spherical joints. The axis of *R*_2_ intersects with this link forming a center, which is deemed to coincide with the pelvic center. Therefore, a body coordinate frame is attached to this center to specify the pelvic motion.

In terms of motion function, as explained before, the four-bar parallelogram mechanism will provide the pelvis with displacement along *X*-axis or *Y*-axis. Since it is a parallelogram, its middle point that is connected to the axis of *R*_2_ follows a circular path. For this reason, the parallelogram is modeled as a revolute joint, and the translations of this point can be expressed as *l*_1_sin*θ*_1_ in the *X*-axis and *l*_1_(1 − cos*θ*_1_) in the *Y*-axis. The six-bar mechanism is also a parallelogram. Its middle point is in line with the pelvic center and follows a circular path, hence providing the rotation around the *Z*-axis. *R*_2_ is used to provide the rotation about the *Y*-axis. The pelvic rotation about the *X*-axis is realized through *R*_4_. In what follows, kinematic modeling of our robot is provided.

## 3. System Modeling

### 3.1. Kinematic Modeling

A robot coordinate frame *oxyz* is attached to the base of the mobile platform with the origin at the center of the vertically mounted linear motor and OXYZ is the world coordinate system (WCS). The *z* axis is along the guideway of the linear motor, the *y* axis is along the walking direction, and the *x* axis is determined by the right hand rule. The patient is considered as the end-effector attached to the center of *R*_4_, to which a body coordinate frame *O*_b_*X*_b_*Y*_b_*Z*_b_ is attached, with origin *O*_b_ at the center of the pelvis. The initial orientation of the body coordinate frame is aligned with the fixed coordinate frame. Vector **P** expresses the position from *o* to *O*_b_. Matrix **R** describes the rotation from the body frame to the fixed frame. By following the motion sequences of the pelvis, the rotation matrix **R** is expressed as

(1) $$\begin{array}{c} R=R\left(Y,\beta \right)R\left(Z,\gamma \right)R\left(X,\alpha \right). \end{array}$$

In this paper, a vectorial method [16] is used for kinematic modeling due to its simplicity. The position vector **P** and rotation matrix **R** are expressed as

(2) $$\begin{array}{c} P=\overset{n}{\underset{i=0}{\sum }}R_{i}P_{i}^{\prime },\\ R=\overset{n}{\underset{i=0}{\prod }}R_{i}, \end{array}$$

where **P**_*i*_′ is the position vector from the *i*th joint to the (*i*+1)th joint in the *i*th local frame, **R**_*i*_ is the rotation matrix from the *i*th frame to the (*i*–1)th frame, and it can be expressed here as **R**_*i*_=**R**_si_**R**_mi_ with matrix **R**_si_ representing the initial set-up between each adjacent frames and matrix **R**_mi_ representing the rotation of the current frame.

After formulating the position vector and rotation matrix as shown in Appendix, **R** and **P** can be combined to form a conventional homogeneous transformation matrix in the joint space as

(3) $$\begin{array}{c} \left[\begin{array}{cc} R & P\\ 0 & 1 \end{array}\right]=\left[\begin{array}{cccc} \text{C}2\text{C}3 & \text{S}2\text{S}4-\text{C}2\text{C}4\text{S}3 & \text{C}4\text{S}2+\text{C}2\text{S}3\text{S}4 & -l_{1}\text{S}1\\ \text{S}3 & \text{C}3\text{C}4 & -\text{C}3\text{S}4 & 0.6+l_{1}\text{C}1\\ -\text{C}3\text{S}2 & \text{C}2\text{S}4+\text{C}4\text{S}2\text{S}3 & \text{C}2\text{C}4-\text{S}2\text{S}3\text{S}4 & z_{0}\\ 0 & 0 & 0 & 1 \end{array}\right], \end{array}$$

where C1 and S1 denote sin*θ*_1_ and cos*θ*_1_, respectively, and the same denotation applies to the other joints. In the task space, the pose of the pelvis is expressed using the following homogeneous transformation matrix in terms of the rotation and translation as

(4) $$\begin{array}{c} \left[\begin{array}{cc} R & P\\ 0 & 1 \end{array}\right]=\left[\begin{array}{cccc} \text{C}\beta \text{C}\gamma & \text{S}\beta \text{S}\alpha -\text{C}\beta \text{C}\alpha \text{S}\gamma & \text{C}\alpha \text{S}\beta +\text{C}\beta \text{S}\gamma \text{S}\alpha & p_{x}\\ \text{S}\gamma & \text{C}\gamma \text{C}\alpha & -\text{C}\gamma \text{S}\alpha & p_{y}\\ -\text{C}\gamma \text{S}\beta & \text{C}\beta \text{S}\alpha +\text{C}\alpha \text{S}\beta \text{S}\gamma & \text{C}\beta \text{C}\alpha -\text{S}\beta \text{S}\gamma \text{S}\alpha & p_{z}\\ 0 & 0 & 0 & 1 \end{array}\right]. \end{array}$$

By equating Equations (3) and (4), the following five equations can be found to solve the inverse kinematics, i.e., solving for the joint variables *q*_*i*_(*i*=0,1,…, 4) under given pelvis movement, that is,

(5) $$\begin{array}{c} z_{0}=p_{z},\\ \theta_{1}=\sin^{-1}\left(\frac{-p_{x}}{l_{1}}\right),\\ \theta_{2}=\beta ,\\ \theta_{3}=\gamma ,\\ \theta_{4}=\alpha . \end{array}$$

The next step is to derive the Jacobian matrix that is required for static modeling. For this purpose, let the linear and angular velocity of the pelvis be ${\left[\begin{array}{cc} \upsilon & \omega \end{array}\right]}^{\text{T}}$, where $\left[\upsilon \right]={\left[\begin{array}{ccc} \upsilon_{x} & \upsilon_{y} & \upsilon_{z} \end{array}\right]}^{\text{T}}$ and $\left[\omega \right]={\left[\begin{array}{ccc} \omega_{x} & \omega_{y} & \omega_{z} \end{array}\right]}^{\text{T}}$. For the prismatic joint ${\left[\begin{array}{cc} \upsilon & \omega \end{array}\right]}^{\text{T}}={\left[\begin{array}{cc} z_{i} & 0 \end{array}\right]}^{\text{T}}{\dot{q}}_{i}$, and for the revolute joint υωT=zi×pin0ziTq˙i. By defining all the components for the five joints, the following velocity relationship is obtained:

(6) $$\begin{array}{c} \left[\begin{array}{c} \nu_{x}\\ \nu_{y}\\ \nu_{z}\\ \omega_{x}\\ \omega_{y}\\ \omega_{z} \end{array}\right]=\left[\begin{array}{ccccc} 0 & -l_{1}\text{C}1 & 0 & 0 & 0\\ 0 & -l_{1}\text{S}1 & 0 & 0 & 0\\ 1 & 0 & 0 & 0 & 0\\ 0 & 0 & 0 & 0 & 1\\ 0 & 0 & 1 & 0 & 0\\ 0 & 0 & 0 & 1 & 0 \end{array}\right]\left[\begin{array}{c} {\dot{z}}_{0}\\ {\dot{\theta }}_{1}\\ {\dot{\theta }}_{2}\\ {\dot{\theta }}_{3}\\ {\dot{\theta }}_{4} \end{array}\right], \end{array}$$

from which the Jacobian matrix of the pelvic support mechanism is given as

(7) $$\begin{array}{c} J=\left[\begin{array}{ccccc} 0 & -l_{1}\text{C}1 & 0 & 0 & 0\\ 0 & -l_{1}\text{S}1 & 0 & 0 & 0\\ 1 & 0 & 0 & 0 & 0\\ 0 & 0 & 0 & 0 & 1\\ 0 & 0 & 1 & 0 & 0\\ 0 & 0 & 0 & 1 & 0 \end{array}\right]. \end{array}$$

Note that this Jacobian is a 6 × 5 matrix, because velocity *v*_*x*_ and *v*_*y*_ are coupled, both generated by ${\dot{\theta }}_{1}$.

### 3.2. Joint Stiffness Modeling

As mentioned before, all the joint compliances are not torsional. Modeling is needed to convert them to torsional stiffness. First, *R*_1_ is attached to a leaf spring. As shown in Figure 3, when *R*_1_ rotates by *θ*_1_, the distance of between two contact points *A* and *B* decreases and the leaf spring is squeezed to generate force *F*_1_ on *A* and *B*, respectively, which can be expressed as

(8) $$\begin{array}{c} F_{1}=k_{1}\Delta_{1}, \end{array}$$

where Δ_1_=*l*_0_(1 − C1) is the squeezed distance, and *k*_1_ is the stiffness of the leaf spring. The difference between moment arm *l*_11_ and *l*_12_ will generate torque *τ*_1_ on *R*_1_, which can be expressed

(9a) $$\begin{array}{c} \tau_{1}=F_{1}\left(l_{11}-l_{12}\right). \end{array}$$

Since (*l*_11_ − *l*_12_)=*l*_0_S_1_, substituting Equation (8) in Equation (9a) leads to

(9b) $$\begin{array}{c} \tau_{1}=k_{1}l_{0}^{2}\left(1-{\text{C}}_{1}\right){\text{S}}_{1}. \end{array}$$

Apparently, Equation (9b) is nonlinear. For the piecewise linear method to be introduced later in the paper, segmentation will be considered. Therefore, for small angle and using sin^2^*α*=(1 − cos2*α*)/2 and sin*α* ≈ *α*, Equation (9b) can be simplified as

(10) $$\begin{array}{c} \tau_{1}=K_{1}\theta_{1}, \end{array}$$

where

(11) $$\begin{array}{c} K_{1}=\frac{k_{1}l_{0}^{2}\theta_{1}^{2}}{2}. \end{array}$$

For *R*_2_ and *R*_3_, linear springs are used, as shown in Figure 3. Considering *R*_2_, there are two linear spring associated with it. With a rotation by *θ*_2_, the two springs generate a deformation Δ_2_ resulting in a force *F*_2_=*k*_2_Δ_2_. Then, torque *τ*_2_ on joint 2 can be expressed:

(12a) $$\begin{array}{c} \tau_{2}=F_{2}L_{k2}, \end{array}$$

where *L*_*k*2_ is the distance between the springs and the axis of joint 2. Note that Δ_2_=*L*_*k*2_tan*θ*_2_. Substitution of *F*_2_ and Δ_2_ to Equation (12a) will lead a nonlinear equation. If segmentation is used, then, Equation (12a) can be simplified as

(12b) $$\begin{array}{c} \tau_{2}=K_{2}\theta_{2}, \end{array}$$

where *K*_2_=*k*_2_*L*_*k*_2__^2^. Likewise, for the linear springs installed on joint 3, the equivalent torsional stiffness is expressed as *K*_3_=*k*_3_*L*_*k*_3__^2^.

### 3.3. Static Modeling

Now, we are ready to derive the relation between the interaction forces and the pelvic movements. Let **C**(*q*) be the compliance matrix of the end-effector of the pelvic support mechanism, then the reaction force **F** on the pelvis can be related to the pelvic displacement **D** as

(13) $$\begin{array}{c} F=C^{-1}\left(q\right)D, \end{array}$$

where $F={\left[\begin{array}{cc} f & n \end{array}\right]}^{\text{T}}$ is the generalized force vector, **f** is the force vector, and **n** is the moment vector. The joint forces can be related to the generalized force vector as

(14) $$\begin{array}{c} \tau =J^{\text{T}}\left(q\right)F, \end{array}$$

where **τ** is the vector containing the joint forces or torques, and **J** is the robot Jacobian derived before. In general, **τ**=*K*_*q*_Δ and **D**=**J**(*q*)Δ where Δ is the vector of the joint deformations, and *K*_*q*_=diag(*K*_1_, *K*_2_,…, *K*_*n*_) is the joint stiffness matrix. From (13) and (14), the generalized compliance matrix for the pelvic support mechanism can be derived as

(15) $$\begin{array}{c} \text{C}\left(\text{q}\right)=JK_{q}^{-1}J^{\text{T}}. \end{array}$$

The system stiffness matrix is the inverse of the compliance matrix above, that is,

(16) $$\begin{array}{c} {\left(JK_{q}^{-1}J^{\text{T}}\right)}^{-1}={\left(J^{\text{T}}\right)}^{-1}KJ^{-1} \end{array}$$

As shown in Equation (7), the Jacobian matrix of the pelvic support mechanism is a rectangular matrix; hence, a generalized inverse is applied to both *J* and *J*^T^ as

(17) $$\begin{array}{c} {\left(J^{\text{T}}\right)}^{+}=J{\left(J_{5\times 6}^{\text{T}}J_{6\times 5}\right)}^{-1}, \end{array}$$

(18) $$\begin{array}{c} {\left(J\right)}^{+}={\left(J_{5\times 6}^{\text{T}}J_{6\times 5}\right)}^{-1}J^{\text{T}}. \end{array}$$

By substituting Equations (17) and (18) back in Equation (13), the relation can be given as

(19) $$\begin{array}{c} \mathrm{F=}J{\left(J^{\text{T}}J\right)}^{-1}K_{q}{\left(J^{\text{T}}J\right)}^{-1}J^{\text{T}}D. \end{array}$$

Then by substituting Jacobian Equation (7) and the stiffness of all the springs into Equation (19), the relation between the pelvic movements and the reaction force can be obtained through symbolic computation as

(20a) $$\begin{array}{c} F=\left[\begin{array}{cccccc} \frac{k_{1}l_{0}^{2}\theta_{1}^{2}{\text{C}}_{1}^{2}}{2l_{2}^{2}} & \frac{k_{1}l_{0}^{2}\theta_{1}^{2}\text{C}1\text{S}1}{2l_{2}^{2}} & 0 & 0 & 0 & 0\\ \frac{k_{1}l_{0}^{2}\theta_{1}^{2}\text{C}1\text{S}1}{2l_{1}^{2}} & \frac{k_{1}l_{0}^{2}\theta_{1}^{2}{\text{S}}_{1}^{2}}{2l_{1}^{2}} & 0 & 0 & 0 & 0\\ 0 & 0 & k_{0} & 0 & 0 & 0\\ 0 & 0 & 0 & 0 & 0 & 0\\ 0 & 0 & 0 & 0 & k_{2}L_{k2}^{2} & 0\\ 0 & 0 & 0 & 0 & 0 & k_{3}L_{k3}^{2} \end{array}\right]\left[\begin{array}{c} \Delta x\\ \Delta y\\ \Delta z\\ \Delta \theta_{x}\\ \Delta \theta_{y}\\ \Delta \theta_{z} \end{array}\right]. \end{array}$$

Equation (20a) indicates a number of points by referring back to Figure 3. First, the stiffness in the *X* and *Y* axis are coupled by *θ*_1_. The stiffness in the *Z*-axis is determined by the vertically mounted linear motor. This motor is being developed with a force control to provide stiffness denoted by *k*_0_. The angular stiffness about the *Y*-axis is provided by joint 2 for lateroflexion. The angular stiffness about the *Z*-axis is provided by joint 3 for pelvic rotation. The bending about the *X* axis is not controlled, as it is formed by the two spherical joints to physically attach the human pelvis to the robot. Therefore, Equation (20a) can be simplified as

(20b) $$\begin{array}{c} F=K_{\text{G}}\Delta X, \end{array}$$

where **F**=[*F*_*x*_, *F*_*y*_, *F*_*z*_, *M*_*y*_, *M*_*z*_]^T^, Δ**X**=[Δ*X*, Δ*Y*, Δ*Z*, Δ*θ*_*y*_, Δ*θ*_*z*_]^T^, and

(20c) $$\begin{array}{c} K_{G}=\left[\begin{array}{ccccc} \frac{k_{1}l_{0}^{2}\theta_{1}^{2}{\text{C}}_{1}^{2}}{2l_{2}^{2}} & \frac{k_{1}l_{0}^{2}\theta_{1}^{2}\text{C}1\text{S}1}{2l_{2}^{2}} & 0 & 0 & 0\\ \frac{k_{1}l_{0}^{2}\theta_{1}^{2}\text{C}1\text{S}1}{2l_{1}^{2}} & \frac{k_{1}l_{0}^{2}\theta_{1}^{2}{\text{S}}_{1}^{2}}{2l_{1}^{2}} & 0 & 0 & 0\\ 0 & 0 & k_{0} & 0 & 0\\ 0 & 0 & 0 & k_{2}L_{k2}^{2} & 0\\ 0 & 0 & 0 & 0 & k_{3}L_{k3}^{2} \end{array}\right]. \end{array}$$

With Equation (20b) formulated, we can now look at how the pelvic movement can be absorbed by the compliant joints. This problem can be stated as given pelvic movement Δ**X** to solve the pelvis reaction force **F**, which can then be related to the joint forces *τ* using Equation (14).

A straightforward ways to determine **F** would be to directly apply Equation (20b). We call this method a linear approach, as it only provides the first order approximate solution assuming **K**_G_ not changing with the pelvic movement. This is not the case, as **K**_G_ is nonlinear, changing with the pelvis movement as a function of the joint angles. Therefore, a piecewise linear method is proposed here to discretize a given Δ**X** into a series of small Δ**X**_*i*_ to account for the change in **K**_G_, then Equation (20b) becomes

(20d) $$\begin{array}{c} F=\overset{N}{\underset{i}{\sum }}K_{\text{G}i}\Delta X_{i}, \end{array}$$

where *N* is the number of segments.

## 4. Simulation and Experiment

**Figure 4:**
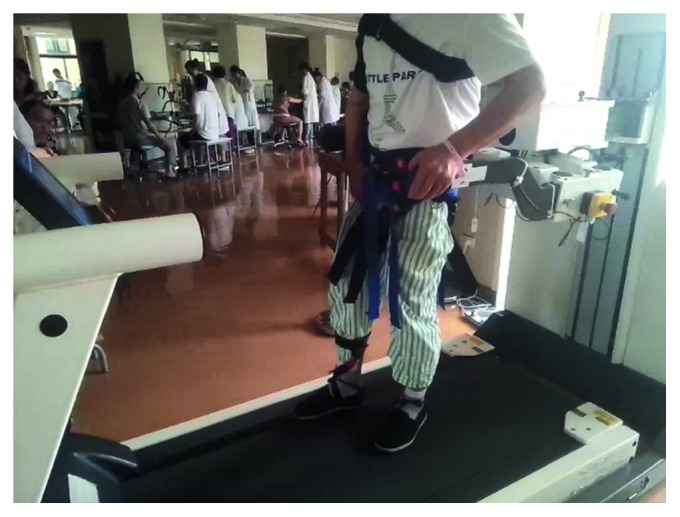
The PB and its mechanical interface with the user.

**Figure 5:**
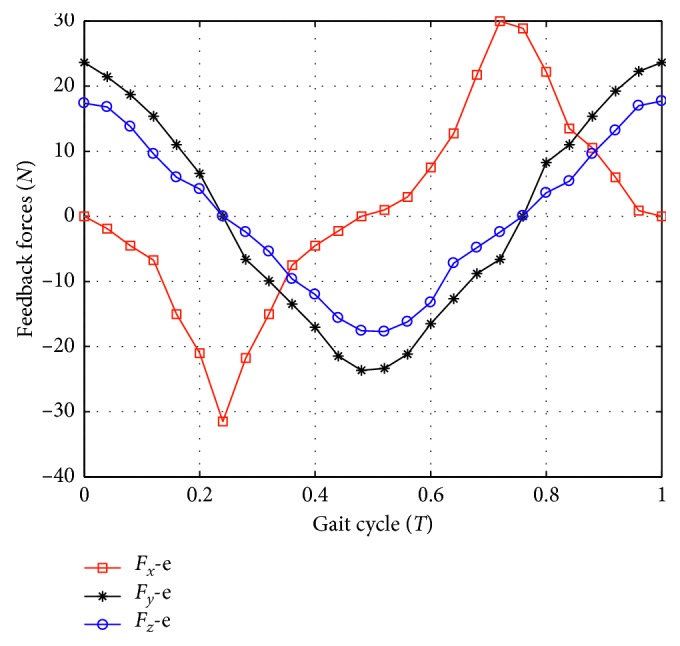
The measured data of the interaction forces on the pelvis.

Simulation and experiment are both carried out to verify the proposed method in chapter III. Parameters of simulation are experimentally determined prior to our research and listed in Table 1. Presented simulations are developed in MATLAB, and pelvic motions are determined by motion analysis of pelvis.  Figure 4 shows a patient with the robot. Two ATI force/torque sensors are connected to both sides of the pelvis to measure the reaction forces. As mentioned before, three joint encoders are embedded in compliant joints 1, 2, and 3 to measure the joint angles. For this experiment, a gait cycle was divided into 25 segments, so in total, 26 points were measured. The experiment was repeated a number of times and the averaged values are shown in Figure 5. Furthermore, three preliminary gait experiments are performed to investigate the effects of the interaction forces and pelvic motions with the RAPBT through force and gait analysis.

### 4.1. Simulation of Linear and Piecewise Linear Approach

**Figure 6:**
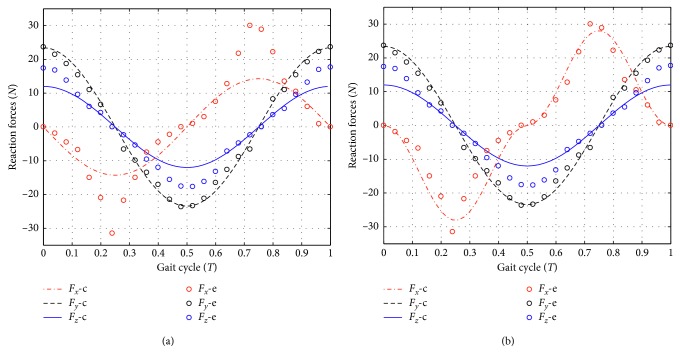
The simulation results of interaction forces with the linear method and the piecewise linear method.(a) The linear approach. (b) The piece-wise linear approach.

In this case, Equation (20b) is directly used for simulation to determine the reaction forces when the mechanism rotates from the original position *q*^T^=[0,0,…, 0]. For joint 1, the stiffness varies with the joint angle changes; therefore, the averaged stiffness from −*θ*_1_ to *θ*_1_is used for linear approach.  Figure 6(a) shows the comparison between the simulation results with the experiment results. It can be seen that the simulation force *F*_*y*_-c and *F*_*z*_-c match with the experiment result *F*_*y*_-e and *F*_*z*_-e fairly well. However, there is a large discrepancy between the simulation force *F*_*x*_-c and the experiment force *F*_*x*_-e mainly because of the nonlinearity in the stiffness of joint 1. Therefore, the linear approach of the pelvic support mechanism is not accurate, as expected at beginning.

In this case, Equation (20d) is used to solve the reaction forces. The final forces are the summation of the segmented forces.  Figure 6(b) shows the comparison between the simulation results with the experiment results. It can be seen that the simulation forces *F*_*x*_-c, *F*_*y*_-c, and *F*_*z*_-c all match well with the experiment result *F*_*x*_-e, *F*_*y*_-e, and *F*_*z*_-e. One may notice that there exists a small discrepancy between forces *F*_*z*_-c and *F*_*z*_-e in Figure 6. This is due to the estimated stiffness value for the linear motor, which is irrelevant of the piecewise linear approach. Therefore, we can state that the proposed piecewise linear method indeed enhances the accuracy of the force determination.

### 4.2. Experiment Design

Ten healthy young adults with no known neurological or orthopaedical disorders, human body weight (60.25 ± 11.55 kg), body height (1.68 ± 0.14 m), and age (26.0 ± 2.23 m) were recruited for this study. All subjects' gait was normal through gait analysis. Motion capture system-OptiTrack (eight Prime 41) was employed to capture the motions of two legs; reflective markers were attached to the right iliac crest, left iliac crest, sacrum, heels, and toes of each volunteer; three Tamagawa encoders were used to obtain the pelvic motions and two ATI force/moment sensors were used to record the interaction force/moment.

All subjects were given informed consent in accordance with Institutional Review Board standards and were instructed to walk naturally on 5 m distance walk way in the laboratory, and gait velocity was determined as required. Three successful trials for each condition were collected for further analysis.

The first experiment was derived out to study the effect of gait velocity on the interaction forces. All subjects were instructed to walk naturally on 5 m distance with gait velocity at the speed of 0 m/s (walking on a treadmill with PB, the velocity relative to the WCS), 0.4 m/s and 0.8 m/s, respectively. The curves applied to the user are the average curves from 10 subjects and the last two tests are performed on ground with PB. The interaction forces *F*_*x*_, *F*_*y*_, and *F*_*z*_ are the output signals from the ATI force sensors. The second protocols were comprised of walking with RAPBT (WR), walking with RAPBT and PB (PB), and walking with PB and getting rid of forces generated by the springs from force inputs in admittance control (WQ). Maximum of forces, mean forces, and scope of pelvic motions were collected to demonstrate effect of PB on the interaction forces and range of pelvic motions. The average value and standard deviation over the different subjects were used to demonstrate the difference. The third protocols were comprised of walking without RAPBT (normal walking, NW), walking with RAPBT without PB (WR), and walking with PB (PB). Normalized stride and step length, step width, gait velocity, lateral displacement, and rotation were collected to demonstrate effect of PB on the gait parameters.

### 4.3. Data Analysis and Discussion

The original data were preprocessed through customized software provided by the control unit (Beckhoff PLC CX5130), and the information of three-dimensional markers was transformed into the gait parameters. The original data of three encoders were transformed into the lateral displacement of pelvis, pelvic tilt, and rotation according to the kinematic model. The force signals from the ATI sensors were preprocessed through the control unit and transformed into maximum and mean value through MATLAB for further analysis and interpretation. All data were analyzed with statistical software SPSS 22.0, and significant differences were indicated with ^*∗*^ for *p* ≤ *α*(0.05) and with a ^*∗∗*^ for *p* ≤ *α*(0.01).

The simulation results of interaction forces with the linear and the piecewise linear method are shown in Figure 6. In comparison to the results of the linear method, the theoretical results of piecewise linear method are verified by the experiments and are more accurate than that of the linear method.

**Figure 7:**
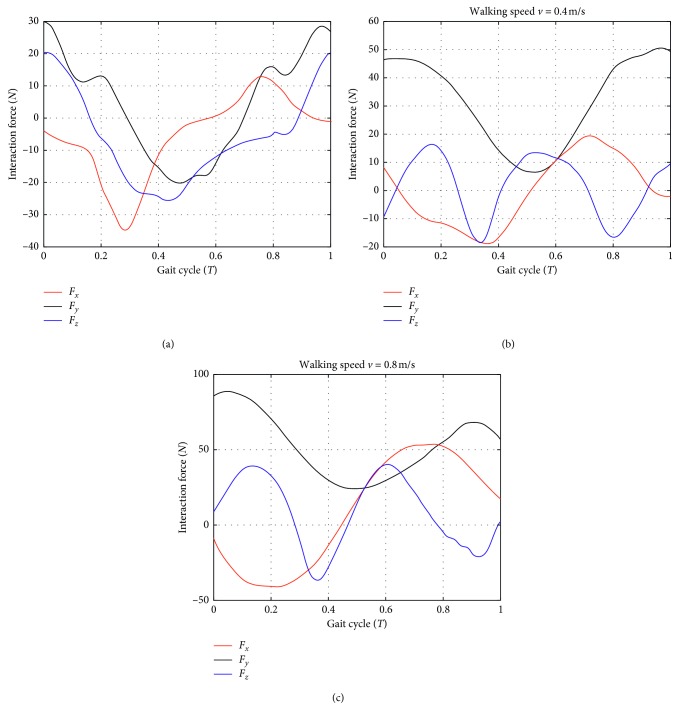
The effect of gait velocity on the interaction forces. From left to right: 0 m/s (walking on a treadmill with PB), 0.4 m/s, and 0.8 m/s, respectively. The curves applied to the user are the average curves from 10 subjects and the last two tests are performed on ground with PB. The interaction forces *F*_*x*_, *F*_*y*_, and *F*_*z*_ are the output signals from the ATI force sensors.

The effect of gait velocity on the interaction forces is shown in Figure 7. It can be seen that the experimental results and simulation results of the piecewise linear method have a similar variation rule; however, the amplitude of interaction forces increases with the gait velocity increase, the variations of *F*_*y*_ is obvious, which indicates that the control of robot needs to improve. When subjects walk on the ground with PB, accelerated velocity of CoM has an effect the interaction forces, and vertical movement of CoM dominate the interaction force *F*_*z*_ and the moment generated by the springs has little effect on it. One of the interesting findings is that the curve of *F*_*y*_ on treadmill is not smooth, the possible reason may be the “*∞*” motion of pelvis.

**Figure 8:**
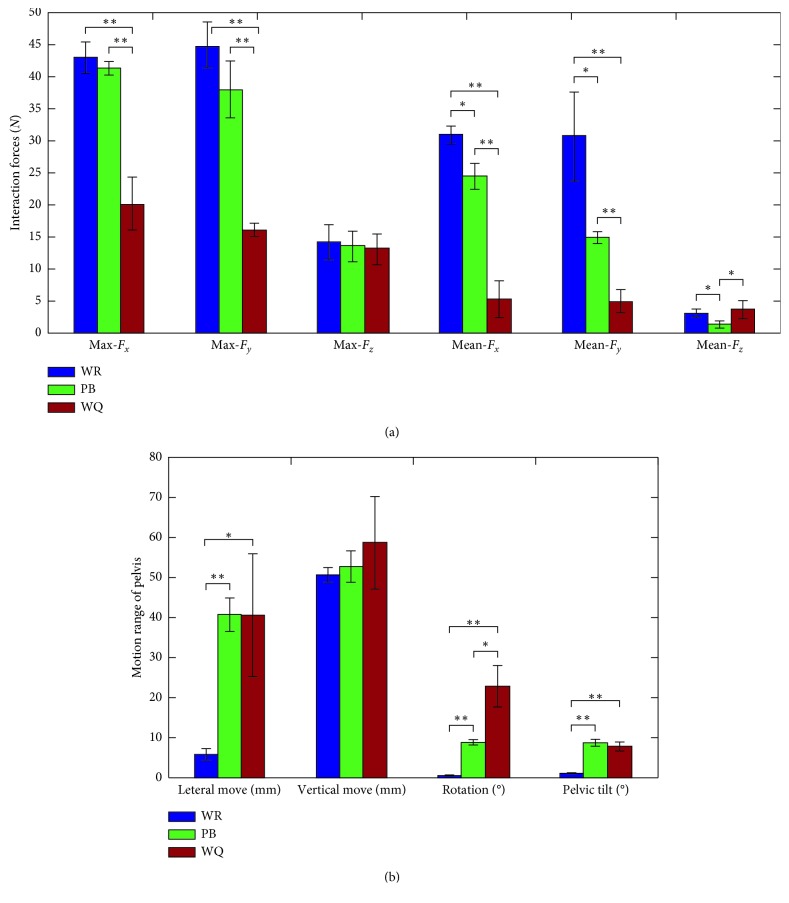
The effect of PB on the interaction forces and motion range of pelvis. Vertical bars indicate the average value and standard deviation over the different subjects. Significant difference between RAPBT walking (WR), RAPBT walking with PB (PB), and RAPBT walking with PB and admittance control (WQ) are indicated with a ^*∗*^ for *p* ≤ *α*(0.05) and with a ^*∗∗*^ for *p* ≤ *α*(0.01).

Figure 8 shows the effect of PB on the interaction forces and range of pelvic motions. It can be clearly seen that WQ can significantly decrease the forces *F*_*x*_ and *F*_*y*_ (maximum and mean value), the possible reason is that the admittance control reduces the influence of inertia, which indicates that WQ is beneficial to the following performance of the RAPBT. Comparing PB with WR, the mean value of *F*_*y*_ is relatively small, the mean value of *F*_*x*_ and *F*_*y*_ are significantly different, PB absorbs part of impact force caused by the accelerated velocity of CoM, increasing the flexibility of the robot and decreasing the impact force. For pelvic motions, WR has a significantly restrictions on pelvic rotation, pelvic tilt, and lateral motion of pelvis. In contrast, PB can satisfy all pelvic motions during gait, but the rotation value has a significantly difference compared with WQ.

**Figure 9:**
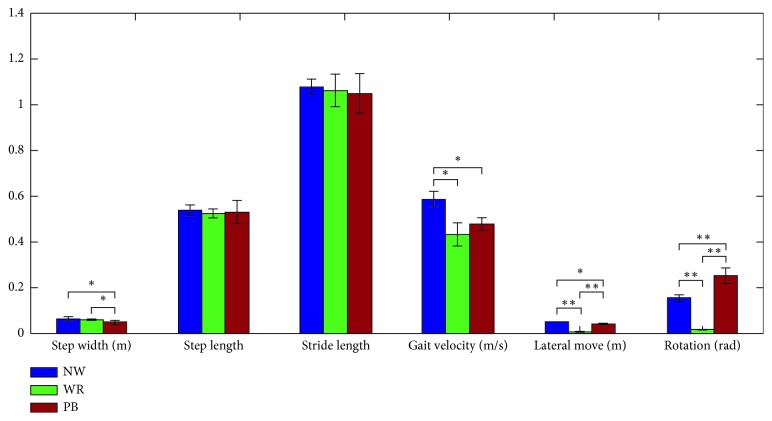
Gait performance parameters such as normalized stride and step length, step width, gait velocity, lateral displacement, and rotation. The experiments were performed in three different conditions: walking without RAPBT (NW), walking with RAPBT while PB was unavailable (WR), and walking with RAPBT while PB was available (PB). Significant difference between NW, WR, and PB are indicated with a ^*∗*^ for *p* ≤ *α*(0.05) and with a ^*∗∗*^ for *p* ≤ *α*(0.01).

Gait performance parameters such as normalized stride and step length, step width, gait velocity, lateral displacement, and rotation in three conditions as mentioned before are shown in Figure 9. On the whole, walking with the robot may influence the gait parameters, but the influence of PB is not obvious. Comparing WR with NW, WR restricts gait velocity and pelvic motion to some extent, and it may be not beneficial to nature gait, which indicates that WR is suitable for the early stage patients. Comparing NW with PB, gait performance parameters are similar, while walking with PB decreases step width, gait velocity, and pelvic motions to some extent, the possible reason may be the effect of interaction force. It is important to those stroke survivors who cannot control the pelvic motions, the force field generated by the PB plays an important role in protection and motor learning.

## 5. Conclusions

A complete force analysis method is provided in this paper to facilitate the introduction of compliance to be embedded in the joints of a pelvic support walking robot. A model between the pelvic reaction force and the pelvic motions is established. This model can be used to determine the joint stiffness and facilitate the control of robot for training injury prevention. If the joint compliance is made adjustable, the same model can be used to determine the joint stiffness to provide different levels of the reaction forces for different stages of training. The key to this success is the piecewise linear method proposed to solve the force problem under given pelvic movement by taking into account the nonlinearity in the system stiffness matrix. The simulation and experiment conducted demonstrate the effectiveness of our method.

The findings of this research demonstrate that gait training with RAPBT strongly resembled free overground walking, while gait with PB led to gait performances with reduction in the range of motion of lower limb. Various training functions such as assistance and resistance, BWS, and lateral balance training approaches will be implemented through advanced control systems, and clinical evaluation with patients will be conducted in the future.

## Figures and Tables

**Table 1:** Parameters of pelvic brace.

Parameters	Link 0	Link 1	Link 2	Link 3	Link *k*_2_	Link *k*_3_
Link length *l*_*i*_ (*m*)	0.01	0.23	0.40	0.40	0.025	0.03
Mass *m*_*i*_ (kg)	4.0	12.0	6.0	8.0	0.2	0.2

## Data Availability

The data used to support the findings of this study are available from the corresponding author upon request.

## Appendix

### Rotation Matrix

For a known coordinate system, $A\equiv {\left\{\begin{array}{ccc} x_{a} & y_{a} & z_{a} \end{array}\right\}}^{\text{T}}$ and a body-fixed coordinate system $B\equiv {\left\{\begin{array}{ccc} x_{b} & y_{b} & z_{b} \end{array}\right\}}^{\text{T}}$, the rotation matrix of **B** with respect to **A** can be defined using a set of three unit vectors representing the principal directions of **B** described in terms of the coordinate system **A**:

(A.1) $$\begin{array}{c} R_{\text{s}0}=\left[\begin{array}{ccc} 0 & -1 & 0\\ 1 & 0 & 0\\ 0 & 0 & 1 \end{array}\right],\\ P_{0}^{\prime }=\left[\begin{array}{c} 0\\ 0\\ z_{0} \end{array}\right],\\ R_{\text{s}1}=\left[\begin{array}{ccc} 1 & 0 & 0\\ 0 & 1 & 0\\ 0 & 0 & 1 \end{array}\right],\\ P_{1}^{\prime }=\left[\begin{array}{c} C1l_{1}\\ S1l_{1}\\ 0 \end{array}\right]\\ R_{\text{s}2}=\left[\begin{array}{ccc} 0 & 0 & 1\\ 0 & 1 & 0\\ -1 & 0 & 0 \end{array}\right],\\ R_{\text{m}2}=\left[\begin{array}{ccc} C2 & -S2 & 0\\ S2 & C2 & 0\\ 0 & 0 & 1 \end{array}\right],\\ P_{2}^{\prime }=\left[\begin{array}{c} l_{2}\\ 0\\ 0 \end{array}\right],\\ R_{\text{s}3}=\left[\begin{array}{ccc} 0 & 0 & -1\\ 0 & 1 & 0\\ 1 & 0 & 0 \end{array}\right],\\ R_{\text{m}3}=\left[\begin{array}{ccc} C3 & -S3 & 0\\ S3 & C3 & 0\\ 0 & 0 & 1 \end{array}\right],\\ P_{3}^{\prime }=\left[\begin{array}{c} l_{3}\\ 0\\ l_{2} \end{array}\right],\\ R_{\text{s}4}=\left[\begin{array}{ccc} 0 & 1 & 0\\ 0 & 0 & -1\\ -1 & 0 & 0 \end{array}\right],\\ R_{\text{m}4}=\left[\begin{array}{ccc} C4 & -S4 & 0\\ S4 & C4 & 0\\ 0 & 0 & 1 \end{array}\right],\\ P_{4}^{\prime }=\left[\begin{array}{c} 0\\ 0\\ 0 \end{array}\right],\\ R_{\text{b}}=\left[\begin{array}{ccc} 0 & 0 & -1\\ 0 & 1 & 0\\ 1 & 0 & 0 \end{array}\right]. \end{array}$$
